# Articulation of the Teeth

**Published:** 1892-06

**Authors:** Isaac B. Davenport

**Affiliations:** Paris.


ARTICLE IV.

### ARTICLE IV. ARTICULATION OF THE TEETH.

**An abstract of a paper by Dr. Isaac B. Davenport, Paris.**

The old man who had lost all but two of his teeth and thanked God that those met, had some conception of the value of articulation. Many people with the mouth half full of teeth could not thank God for more masticating surface than this old man had, because their teeth strike together so badly that only a few are capable of mastication. The fact that such are often pretty healthy people does not prove that a very limited masticating surface is not deleterious. I believe the perfect articulation of the teeth is that towards which our operations should be directed.

I have no patience for the dentists who go chopping about the mouth like a bushwacker.

In trying to discover that form of articulation most favorable to its greatest usefulness, permanence and regularity, it became evident that some other method was needed than observation in the mouth, where only the outside of the teeth could be seen in contact, or with whole models from which only an imperfect idea of the inside striking of the teeth could be had.

To overcome some of these difficulties we have devised an articulator with triple hinge, which opens laterally as well as vertically.

Mere antagonism seems to express the prevalent idea of articulation; but antagonism is just what is most objectionable in an articulation. Teeth antagonize when they strike only here and there and prevent the other surfaces from touching. When thus arranged they may punch the food, but they cannot chew it. On the other hand a correct articulation of the teeth secures their very highest masticatory functions. In other words, a perfect articulation is the harmonious adjustment to each other of two most beautifully complicated, uneven, triturating surfaces in such a way as to permit all the movements of mastication, each prominence or depression having special reference to the normal movements of mastication, and to change their form or direction would be to render such movements impossible.

When thus perfectly arranged the masticating surfaces slide on and into each other, constituting a self-sharpening machine, made up of a complicated system of inclines, so balanced and bound together as to be practically permanent.

The forces being thus widely distributed, wear goes on slowly on all the surfaces alike, and the physiological process of pulp protection is seldom overtaken by exposure of sensitive fibrils, as is often when but little masticating surface can be employed.

I was formally under the impression that perfectly articulating teeth would finally wear flat, owing to the shortening of the bite, and the under jaw coming forward, the ends of the incisors and the cusps thus disappearing coincidently; but the perfect adaptation to its function may tend towards the preservation of an organ.

A good articulation is where the arches combine most of the desirable qualities, such as continuous grinding surfaces, good contact for the most part inside and out, extensive contact possible on many planes without interfering with individual prominence; slight overshutting of upper incisors permitting the cutting movement without throwing the back teeth too far out of relation; the lower incisors capable of moving backward on the posterior inclines of the upper incisors, just sufficiently to conduct the cusps of the back teeth into their normal depressions with the same sliding contact. The rotary motion is permitted without the normal extent of contact being disturbed, either by too long cuspids or by such malposition of the teeth as to entangle the cusps, for when a cusp by a false contact interferes, being out of relation with the rest of the surfaces, the movement is either arrested, or if continued, the effect of the rotation is destroyed because one cusp must raise over the other, and that separates the other surfaces.

To resume, we find in a good articulation the arches mutually supporting and tending neither to irregularity, contraction, spreading, separation, anterior projection, nor flattening; wear evenly distributed, aud consequently slow.

I have heretofore endeavored to show how the closed teeth should form one continuous self-supporting arch, "each jaw being," as Dr. Dwinelle once aptly remarked during the discussion of this subject, "a perfect matrix for the other;" yet in conversation with many eminent dentists since that paper appeared, I have not found one who grasped the idea of the cross or binding articulation of the teeth, especially the bicuspids.

The normally arranged human teeth should touch all around in both arches.

To be well articulated the teeth must be regularly placed and correctly inclined.

The most common irregularity of the teeth is the irregularity of the position of the masticating surfaces, yet little attention is given this in works on irregularities, the attention being mostly confined to the deviations of the external curves or alignment of the teeth; yet if the former were attended to, the latter would necessarily be corrected, and more permanently than is usual with ordinary methods.

When teeth are regular and well articulated, they remain so because the forces and resistances are evenly balanced.

On the other hand, as the articulation is made up of a series of perfectly balanced inclines, it follows that when anything removes one surface, whether an extraction, decay, operation, or badly-constructed regulating or other apparatus, improper force falls on other inclines changing the position of the teeth.

A few years after the teeth had been extracted, I have hardly ever found what I could call a good articulation, and the same is true when the teeth have been cut away between, or when the cusps had been carelessly removed while finishing fillings in the grinding surfaces. Such teeth antagonize but do not articulate, and cusps strike cusps, point to point, instead of passing between each other like cogs; and the motions of mastication are interfered with, especially the rotary, which is the essentially grinding motion, and hence the rapid wear of the limited number of antagonizing points goes on.

Largely on account of bad articulation, irregular teeth tend to become more irregular. Growth may improve some, but so far as a bad articulation goes, it is always unfavorable to regularity.

Much harm is done by the use of regulating appliances which change the articulation without improving it, and it is almost a universal fact that, unless an improvement can be made in an articulation, there will be no permanent improvement of the irregularity. The articulation is the only permanent retainer to be depended on. For the teeth will move till they find the best contact that circumstances can offer, and that movement often never ceases, because the forces never find equilibrium.

Before disturbing the articulation of a fixed irregularity, it is best to consider whether such disturbance can be overcome, and the articulation again made as good; if not, the chances are that the result will be worse than the original condition, and for the ultimate result we must wait not only "till the teeth become firm," as we say, but till they cease to move.

There is much yet to learn in regard to the meaning of the elevations, depressions, overshutting, shelving, interlocking, binding, curves, and inclines of the articulation, in their relation to biting, cutting, tearing, crushing and grinding movements of mastication.

In the treatment we may hope that at least articulations are not worse by our operations than when brought to us.

I will renew my indorsement, made before the International Dental Congress at Paris, of the use of Dr. Bonwill's articulator for the arrangement of artificial teeth.—*Items of Interest.*

